# Intestinal Microbiota in Common Chronic Inflammatory Disorders Affecting Children

**DOI:** 10.3389/fimmu.2021.642166

**Published:** 2021-06-07

**Authors:** Anna Torun, Anna Hupalowska, Piotr Trzonkowski, Jaroslaw Kierkus, Beata Pyrzynska

**Affiliations:** ^1^ Chair and Department of Biochemistry, Medical University of Warsaw, Warsaw, Poland; ^2^ Klarman Cell Observatory, Broad Institute of MIT and Harvard, Cambridge, MA, United States; ^3^ Department of Medical Immunology, Medical University of Gdansk, Gdansk, Poland; ^4^ Department of Gastroenterology, Hepatology, Feeding Disorders and Pediatrics, The Children’s Memorial Health Institute, Warsaw, Poland

**Keywords:** chronic inflammatory disorders, pediatric diseases, microbiota, immune homeostasis, adoptive cell therapy, regulatory T cells

## Abstract

The incidence and prevalence rate of chronic inflammatory disorders is on the rise in the pediatric population. Recent research indicates the crucial role of interactions between the altered intestinal microbiome and the immune system in the pathogenesis of several chronic inflammatory disorders in children, such as inflammatory bowel disease (IBD) and autoimmune diseases, such as type 1 diabetes mellitus (T1DM) and celiac disease (CeD). Here, we review recent knowledge concerning the pathogenic mechanisms underlying these disorders, and summarize the facts suggesting that the initiation and progression of IBD, T1DM, and CeD can be partially attributed to disturbances in the patterns of composition and abundance of the gut microbiota. The standard available therapies for chronic inflammatory disorders in children largely aim to treat symptoms. Although constant efforts are being made to maximize the quality of life for children in the long-term, sustained improvements are still difficult to achieve. Additional challenges are the changing physiology associated with growth and development of children, a population that is particularly susceptible to medication-related adverse effects. In this review, we explore new promising therapeutic approaches aimed at modulation of either gut microbiota or the activity of the immune system to induce a long-lasting remission of chronic inflammatory disorders. Recent preclinical studies and clinical trials have evaluated new approaches, for instance the adoptive transfer of immune cells, with genetically engineered regulatory T cells expressing antigen-specific chimeric antigen receptors. These approaches have revolutionized cancer treatments and have the potential for the protection of high-risk children from developing autoimmune diseases and effective management of inflammatory disorders. The review also focuses on the findings of studies that indicate that the responses to a variety of immunotherapies can be enhanced by strategic manipulation of gut microbiota, thus emphasizing on the importance of proper interaction between the gut microbiota and immune system for sustained health benefits and improvement of the quality of life of pediatric patients.

## Introduction

The role of the immune system is to efficiently target diverse pathogens, such as viruses and bacteria, to keep cancer cells in check and avoid reactions against its own tissues and organs (1, 2). Inflammation is the defense mechanism of the body by which the immune system recognizes and removes harmful and foreign stimuli and initiates the healing process (3). There are two types of inflammation: acute and chronic (4). Acute inflammation starts rapidly, becomes severe in a short period of time and lasts for a few days (5), whereas chronic inflammation is slow and lasts for prolonged periods of time–from several months to years. Chronic inflammation can result from a failure in eliminating pathogenic organisms during acute inflammation, prolonged exposure to irritants or foreign materials, defects in the immune system, or autoimmune disorders (6).

During inflammation in response to foreign antigens, the immune cells of the tissue, such as macrophages and dendritic cells, release cytokines [e.g. interleukin-1 (IL-1) and tumor necrosis factor-α (TNF-α)] that stimulate the infiltration of circulating leukocytes (7, 8). In addition to the recruitment of leukocytes, the tissue immune cells also play a role in antigen removal by phagocytosis and serve as antigen-presenting cells (APCs) to lymphocytes (9). Neutrophils are the first leukocytes that enter the local injury site. They destroy the antigen by phagocytosis and release granules rich in enzymes, reactive oxygen species, and cytokines, such as IL-1, IL-6, and TNF-α (10, 11). Lymphocytes, including different types of T and B cells, are the next line of defense. They play a crucial role in inflammation by secreting cytokines, producing antibodies and immune complexes (12, 13). The production of inflammatory cytokines, growth factors, and enzymes during inflammation may lead to tissue damage and secondary repair processes (4).

**Figure 1:**
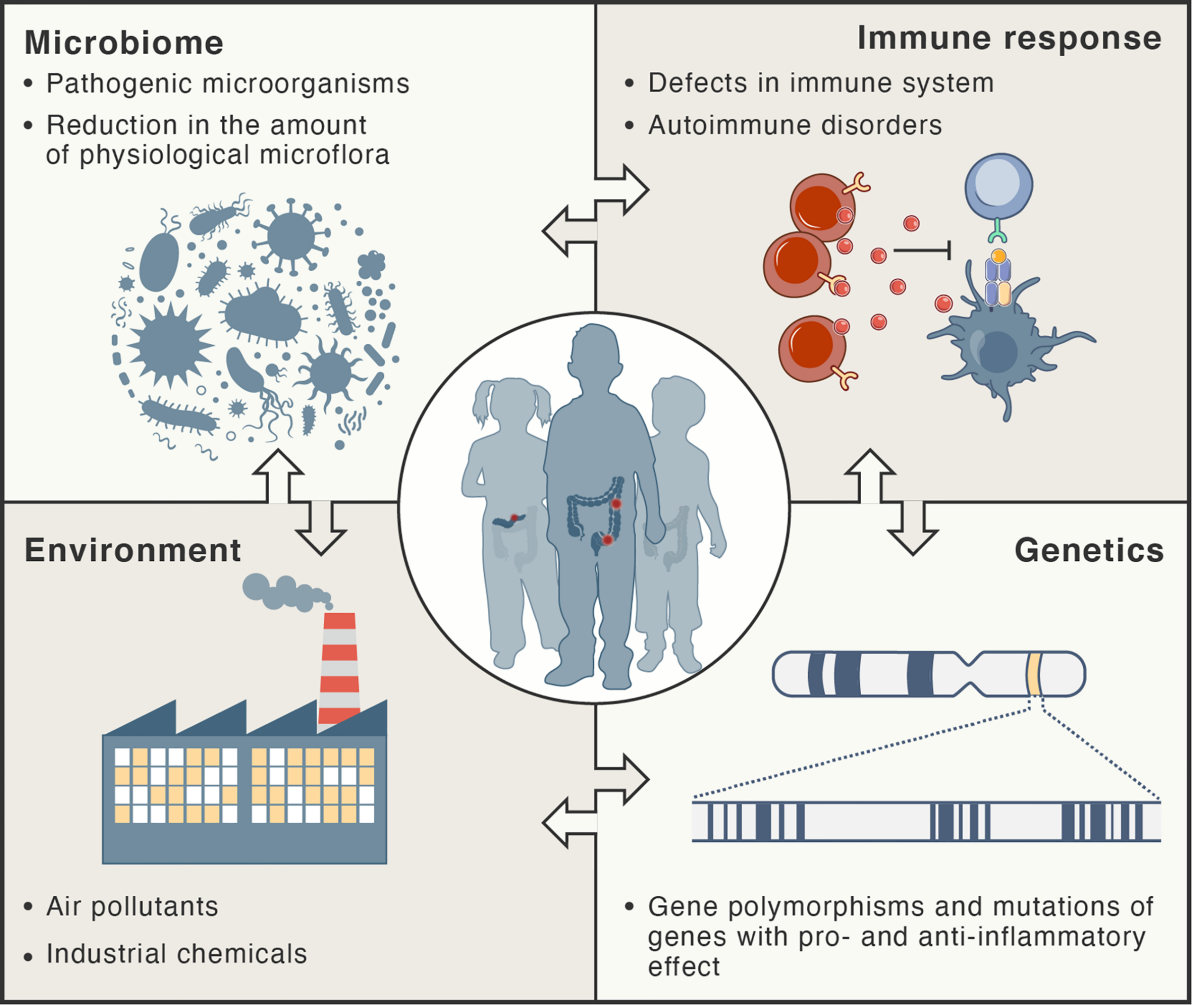
Risk factors for development of chronic inflammatory disorders. These types of diseases are thought to develop as result of complex interactions between the immune system, microbiome, and environment in genetically-susceptible hosts.

Inflammation in autoimmune disorders is distinct; the immune system recognizes the normal components of the body as foreign antigens and attacks healthy tissues (2, 14). Auto-reactive T cells attack and damage specific tissues and organs. Several models for explaining molecular mechanisms triggering autoimmunity have been developed, including molecular mimicry, breach in central tolerance, non-specific bystander activation and persistent antigenic stimuli (15). A recent review provided a comprehensive summary of the concept of molecular mimicry and its potential involvement in different autoimmune diseases (16). The hypothesis of molecular mimicry of foreign antigens by the structures of the body assumes that two different molecules (such as foreign and self-peptide) have antigenic structures similar enough to be recognized by the same antibodies or T cells (antigenic cross-reaction). Regulatory T cells (T_reg_) are the major cell subset maintaining tolerance to self-antigens as they can potently suppress the over-activation of different immune cells, including effector T cells (T_eff_), B cells, natural killer (NK) cells, macrophages, and dendritic cells, and hence, they can maintain the balance between autoimmunity and self-tolerance (2, 17–19). **Figure 1** summarizes factors that participate in the pathogenesis of chronic inflammatory disorders.

In this review, we discuss common chronic inflammatory disorders in children, such as type 1 diabetes mellitus (T1DM), celiac disease (CeD), and inflammatory bowel disease (IBD), with a focus on the role of microbiota in their pathogenesis. We describe current clinical approaches with manipulation of gut microbiota for pediatric therapy. Since many chronic inflammatory disorders arise from an imbalance between T_eff_ cells and T_reg_ cells, we point out the challenges in the development of effective therapies, disabling over-activated T_eff_ cells, as well as the adoptive cell therapies, employing suppressive T_reg_ cells.

## Microbiota

The human intestine harbors approximately 10^13^ to 10^14^ commensal microorganisms, such as bacteria, viruses, and fungi, collectively termed as the microbiota (20). The intestinal microbiota maintains the integrity of the intestinal wall, protects against the overgrowth of pathogenic microorganisms by competing for the same nutrients and synthesizing protective substances, assists in food digestion, and produces vitamins and immunomodulating compounds such as short-chain fatty acids (SCFAs) (21–27). Although, some comprehensive reviews have summarized recent discoveries on the role of microorganisms in human health (28–30), in the following parts, we highlight a few important facts related to changes in microbiota composition and diversity during a life-time.

Microbial colonization of the human gut from maternal (for example, breast milk or birth canal) and non-maternal sources (for example, the diet and environment) and maturation of the gut barrier occur during early life (31–33). The contact with specific microbes during the first 6 months of life is considered as most crucial for the gut maturation (34). Finally, by three years of age, the microbiome profile exhibits the maturity similar to that found in adults (35, 36). Approximately 2000 bacterial species have been isolated from the intestine and most of them belong to four phyla: Firmicutes, Bacteroidetes, Proteobacteria, and Actinobacteria. About 60% of the human gut bacteria belong to the phylum Firmicutes, most of which have gram-positive cell wall structure. They are present mainly in the mucus layer of the intestine. Bacteroidetes, the second most abundant phylum, constitutes about 30% of all bacteria in the gut and are gram-negative bacteria localized primarily in the gut lumen (37–39). The Firmicutes : Bacteroidetes ratio has been considered to be an indicator of the degree of maturation of the human gut microbiota and is lower in infants and the elderly compared to that in adults (40). *Clostridium*, *Streptococcus*, *Ruminococcus*, *Lactobacillus*, and *Bifidobacterium* (gram-positive bacteria) as well as *Bacteroides* and *Escherichia* (gram-negative bacteria) are the most prevalent bacterial genera in the human intestine (41). A recent large cohort study compared the stool microbiome of children and adults, revealing that children exhibit lower microbiota diversity with higher *Bacteroides* abundance and different metabolic pathways (42).

The presence of a large number of symbiotic microorganisms near the epithelial surface is an enormous challenge for the mucosal immune system because it must avoid harmful inflammatory responses to the symbionts, while preserving the ability to mount an immune response against pathogens (43). The interaction between gut microbiota and host cells is regulated by the immune system through pattern recognition receptors, including Toll-like and NOD-like receptors (44). Immunoglobulins (Ig) A and G are the predominant antibody isotypes contributing to intestinal barrier maintenance, microbiome selection, and decreased activation of innate immunity (45, 46). Microbiota-specific IgA and IgG are transmitted to newborns *via* maternal milk, to protect the neonatal intestine from bacterial translocation across the intestinal epithelium (46). Later in life, IgA and IgG are produced at mucosal sites by gut-associated lymphoid tissues (GALTs) and secreted into the intestinal lumen, where they limit the translocation of microorganisms into the body (47–49).

Recent findings associating colonization of the infant intestine by commensal microorganisms with the proper development and maturation of the immune system in mucosal tissues have been comprehensively reviewed (50–53). The early life seems to be a critical period in which immune system education takes place and the immune cells learn to tolerate commensal microbiota. Therefore, perturbed crosstalk between the microbiota and the immune system at this time can lead to serious life-lasting health defects. For example, an elevated risk of developing chronic inflammatory disorders, including T1DM (54, 55), CeD (56) and IBD (57), is associated with microbial dysbiosis in infants. Numerous studies using germ-free (GF) animals have provided insights into the mechanisms, by which the microbiota influences immune system development and maturation [reviewed in Ref (50, 52)]. Very limited exposure of the immune system to appropriate microbiota in early life leads to morphological abnormalities in GALTs, including Peyer’s patches, isolated lymphoid follicles and mesenteric lymph nodes (50). Functional impairment of the mucosal immune system in GF animals is related to decreased quantities of certain T cell subsets, including T_eff_ and T_reg_ cells, and decreased production of anti-microbial IgAs and IgGs. Such defects can be partially restored through gut colonization by a diverse microbial population (58–63). In contrast, the increased accumulation of invariant natural killer T (iNKT) cells in colons of GF animals has been detected and associated with susceptibility to colitis (64, 65). Interestingly, in mice the inhibition of iNKT cell expansion and susceptibility to colitis can be reversed only in the first 2 weeks of life, with the presence of *Bacteroides fragilis* in the colon (64, 65).

### Perturbations in the Human Gut Microbiota

**Figure 2:**
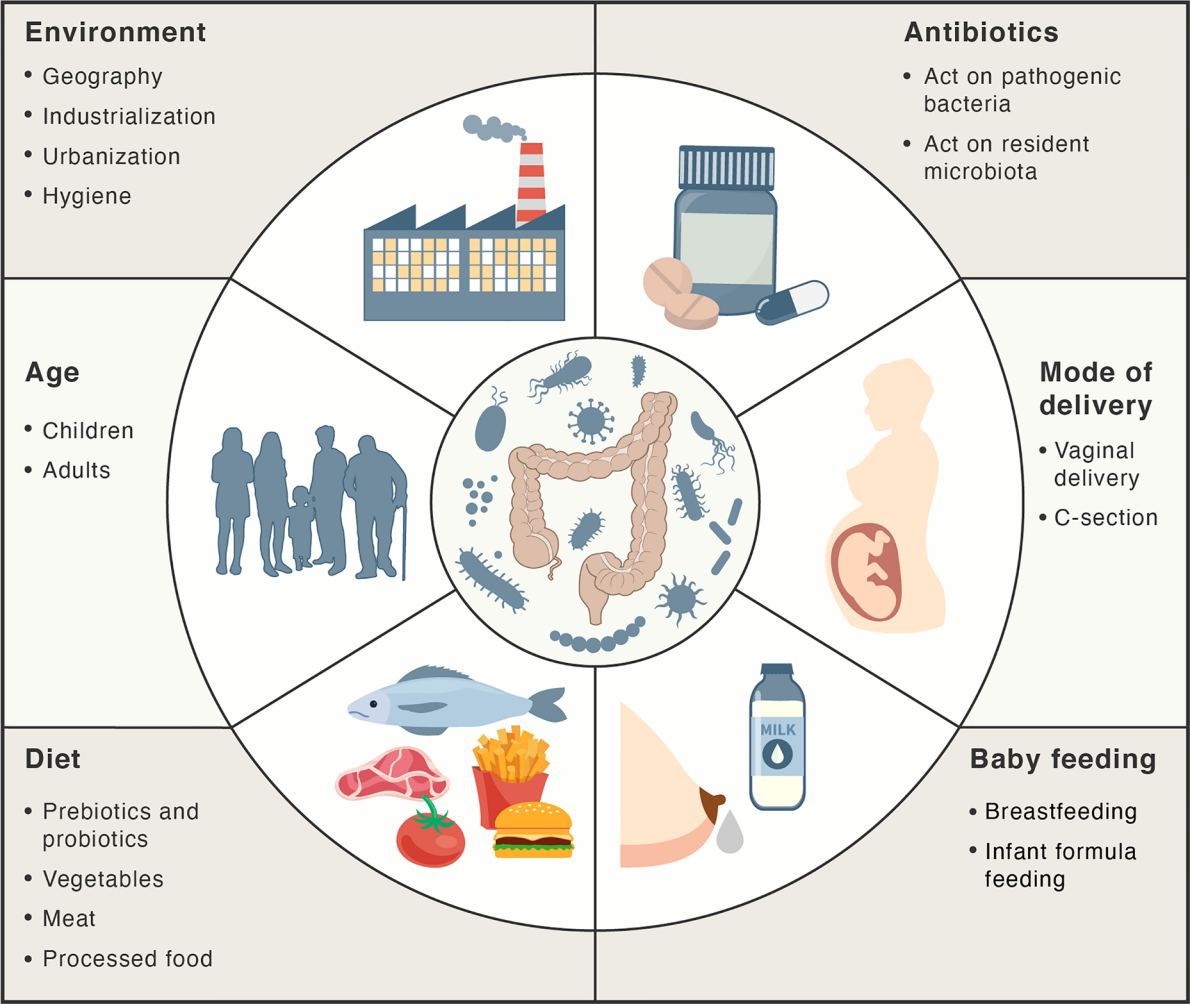
Main factors influencing the gut microbiota. Factors, such as mode of delivery, type of baby feeding, diet, age, environment and antibiotics may act positively or negatively on the intestinal microbiota composition and abundance.

There are several factors that can influence intestinal homeostasis, including cesarean delivery, feeding infant formula instead of breastfeeding, antibiotics, diet, geography, and hygiene (66) (**Figure 2**). A higher degree of gut microbial diversity, with an increased abundance of *Bacteroides*, was observed in infants delivered vaginally than in those by C-sections (67, 68). These differences persisted up to the second year of life and are believed to result from the contact of the infant with vaginal microbiota (for example, *Lactobacillus*) of the mother during birth (68–71). Conversely, C-sections are associated with increased levels of intestinal pathogens such as *Klebsiella*, *Citrobacter*, and *Escherichia coli* (72, 73).

Breastfeeding and antibiotics use are the most significant factors associated with shaping the development of microbiome structure during the first year of life. Breast milk plays a major role in limiting intestinal permeability and establishing a healthy gut barrier, as it contains a variety of nutrients, vitamins, macromolecules, and immunoglobulins (46, 74, 75). Moreover, it contains probiotic bacteria, such as *Lactobacillus rhamnosus*, *L. gasseri*, *Lactococcus lactis*, *Leuconostoc mesenteroides*, and *Bifidobacteria* (76, 77). The microbiota in breast milk induces immune tolerance, prevents infection, and participates in the maintenance of the epithelial barrier (76, 78). The oligosaccharides in breast milk act as prebiotic substrates for bacterial fermentation and contribute to the establishment of the infant gut microbiota (77). SCFAs that are released upon the breakdown of these oligosaccharides maintain intestinal integrity and minimize the growth of pathogenic microorganisms (79). These oligosaccharides have also been shown to inhibit the adhesion of pathogenic bacteria such as *E. coli*, *Vibrio cholerae*, and *Salmonella fyris* to epithelial cells (80). Oligosaccharide concentrations are higher in colostrum on the fourth day post childbirth (preterm mother’s milk) than that in mature milk at thirty days post childbirth, highlighting the importance of breastfeeding during the first days of life (81).

It has been reported that in formula-fed infants the microbiota exhibits a lower abundance of Firmicutes, Actinobacteria, and *Bifidobacterium* compared to breastfed infants (67, 68). Proper gut barrier maturation, manifested by the decrease in intestinal permeability, is particularly important in preterm infants, who are susceptible to necrotizing enterocolitis illness, resulting from a “leaky gut” and dysbiosis (82, 83). Importantly, gut barrier maturation in preterm infants can be induced by exclusive breastfeeding till the tenth day after birth (31, 82). The abundance of the members of Clostridiales in fecal microbiota seems to be associated with early breastfeeding (31). Moreover, breastfeeding decreases the incidence of gastrointestinal tract infections in infants (84).

Antibiotic treatments may also influence the microbiota by providing favorable conditions for the survival and overgrowth of pathogenic microorganisms in the intestine, such as *S. typhimurium* and *Clostridium difficile* (85, 86). It has been shown that oral antibiotics alter the gut microbiota for periods of time ranging from a few weeks to years (87–89). Increased antibiotic use, cleaner living conditions, and urbanization have also changed the exposure to different microorganisms. According to the hygiene hypothesis, decreased exposure to microbial antigens during early life could have a negative effect on the development of the adaptive immune response and may eventually lead to the development of autoimmune disorders. Evidence to support this hypothesis is the observation that both the temporal and geographic incidence of autoimmune and inflammatory diseases is seen to be parallel with the industrialization and urbanization of societies (90–92).

### Modulation of Microbiota Composition

Recent advances in main microbiota-modulating methods have been described in details by Quigley and Gajula (93). The most common interventions include lifestyle modifications, such as diet changes, caloric restriction, and exercise, as well as clinical interventions, such as administration of probiotics, prebiotics, or antibiotics, and fecal microbiota transplantation (93). It is worth underlining that dietary changes, if sufficiently drastic, can rapidly change the gut microbiota, even in the span of 24 hours. Changes in dietary practices usually have long-term effects on the composition and functional capacity of the microbiota (94). It has been reported that diets high in fermentable plant sources lead to an increased abundance of Firmicutes, which metabolize dietary plant polysaccharides. In contrast, high-meat/low-plant diets lead to increased abundance of bile-tolerant microorganisms, such as *Alistipes*, *Bilophila*, and *Bacteroides* species, and a decreased abundance of Firmicutes (95).

Prebiotics, other important microbiota-modulatory factors, have been broadly defined by the International Scientific Association for Probiotics and Prebiotics as “substrates that are selectively utilized by host microorganisms conferring a health benefit” (96). The most commonly used are carbohydrate-based prebiotics, metabolizable by the gut microbiota, leading to selective stimulation of growth or activity of beneficial gut microorganisms. The fermentation products of prebiotics, mainly SCFAs, help to maintain the gut barrier integrity (97, 98).

Probiotics are live microorganisms that provide health benefits upon consumption by improving or restoring the gut flora. They exert their effects by reducing colonization by pathogenic microorganisms, enhancing mucus production, improving epithelium integrity, and balancing the interactions between gut bacteria and the immune system (99–101). In infants who receive probiotics, the changes induced by antibiotic treatment and cesarean delivery are reversed, and the normal composition and function of gut microbiota is restored (102–105).

The last method of modulating gut microbiota that we discuss here is fecal microbiota transplantation (FMT), which involves the transfer of fecal bacteria from a presumptively healthy donor into the gastrointestinal tract of affected patient, in order to treat microbial dysbiosis (106). This method reduces intestinal inflammation, as demonstrated by decreased levels of proinflammatory cytokines such as TNF-α, IL-1β, and interferon-γ (IFN-γ), and helps in restoring the intestinal homeostasis. It has been successfully used for treating recurrent and antibiotic-refractory *C. difficile* infections (107–110), even in pediatric patients (111).

## Type 1 Diabetes Mellitus

T1DM is an autoimmune disease resulting from an inappropriate immune response that causes the destruction of insulin-secreting β-cells of the pancreatic islets, mediated mainly by autoreactive effector T cells [reviewed in (112)]. Destruction of pancreatic β-cells leads to the loss of endogenous insulin production, and results in impaired glucose metabolism. T1DM is usually diagnosed in children or young adults, but it can appear at any age (113, 114). With the increasing incidence of this disorder, the peak age at diagnosis has shifted to a younger age (115).

The major genetic risk factors involved in the development of T1DM are located within the class II human leukocyte antigen (*HLA*) region (116). The most important loci encode HLA-DR and HLA-DQ molecules that bind and present antigenic peptides to T cells. The high degree of polymorphism in the *HLA* region indicates that it is possible to mount an antibody response against a large number of constantly mutating microbial pathogens. Consequently, this wide diversity of recognized epitopes may cause the generation of antigenic cross-reactions due to molecular mimicry. Approximately 70% of T1DM cases carry *HLA* risk alleles (117, 118). The loci identified outside the *HLA* region are associated with polymorphisms of the insulin gene *INS* and lymphocyte protein tyrosine phosphatase *PTPN22* gene, resulting in an increased risk of development of T1DM. A disease-associated genotype of the INS gene is associated with a poor expression of insulin in the thymus, leading to autoreactive INS-specific T cells not being destroyed during the thymic education of T cells (116, 119).

Autoantibodies that recognize insulin, glutamic acid decarboxylase 65, islet antigen 2, and zinc transporter 8 are most often present in the serum of patients with T1DM, and are the best characterized autoantibodies associated with T1DM (120). These autoantibodies are usually present before any dysglycemia or clinical symptoms appear (121). If only one of these major autoantibodies is present, the risk of T1DM is small; however, the presence of two or more autoantibodies indicates a high probability of developing the disease (122). Interestingly, children of mothers with T1DM have a relatively low genetic risk of developing T1DM themselves, as they are at half of the same genetic risk compared to children with a father who has T1DM. This observation shows that islet-specific autoantibodies found in the sera of mothers with T1DM transferred to the fetus do not damage the fetal pancreatic β-cells (123).

The diagnosis of diabetes is based on the measurement of blood glucose concentration and the presence of symptoms (124). Although in the early stages the disease is clinically silent, hyperglycemia and increased production of ketones from fatty acids eventually lead to polydipsia, polyuria, weight loss, and diabetic ketoacidosis (125). The chronic long-term complications associated with the disease include retinopathy, nephropathy, neuropathy, and cardiovascular disease (126, 127). T1DM during puberty appears to accelerate the development of complications (128).

### Role of Gut Microbiota in T1DM

Recently, increasing incidence of T1DM in children within genetically stable populations has been observed (129), with less than 10% of genetically susceptible people developing clinical T1DM (130). This indicates that non-genetic factors also play an important role in the development of T1DM. Among the environmental factors that affect the development of T1DM microbiota composition, microbial infection and nutrition appear to be key factors. Recent reviews have addressed the involvement of dysbiosis in the pathogenesis of T1DM (131–135). Here we very briefly highlight some of the key findings.

Increased permeability of the intestine and changes in the composition of microbiota seem to be associated with T1DM pathogenesis (54, 136–143). Comparison of multiple islet autoantibodies-positive children with healthy controls demonstrated increased abundance of the *Bacteroides* species compared with Firmicutes species (137, 143, 144). The association of *Bacteroides stercoris, B. fragilis, B. intestinalis, B. bifidum* as well as *Synergistetes* taxa with diabetes was recently confirmed by machine-learning analyses of bacterial taxa and their metabolic pathways in children at T1DM onset (145). Autoantibody-positive children exhibit a low abundance of microbiota that produce butyrate (137, 141, 146). It has been reported that butyrate-induced mucin production is important for the integrity of the gut mucosa, and low levels of species that degrade mucin might imply an increased gut permeability (147). Moreover, a reduction in SCFA-producing bacteria was observed in T1DM at the onset of autoimmunity, which is associated with impaired gut barrier functions (148, 149). Decreased diversity of the intestinal microbiome has been reported in pediatric patients with β-cell autoimmunity and in those who progressed to clinical T1DM compared with non-seroconverted controls (137, 141). A marked drop in the diversity of the gut microbiota was also noted in infants genetically predisposed to T1DM during the time window between seroconversion and T1DM diagnosis (54). At the same time, a higher abundance of several pathobionts, including *Ruminococcus gnavus* and *Streptococcus infantarius*, and a lower abundance of bacteria known to counteract inflammation, such as *Lachnospiraceae* and *Veillonellaceae*, were detected in T1DM-affected patients. Moreover, there is a correlation between the changes in the gut microbiota and the pattern of T1DM progression, because the seroconverted group of patients revealed intermediate abundance of all these microorganisms compared to the non-converted and T1DM groups (54).

Some studies showed that delivery by C-section can increase the risk of T1DM in infants (150–153), suggesting that microbiota transfer from the mother’s birth canal to the newborn may be protective against T1DM incidence in infants. Moreover, a higher T1DM risk was observed in children born through planned C-sections than in those born *via* unscheduled C-sections (154). Therefore, the high number of C-sections in developed countries may be partially responsible for the observed increased rate of diabetes (155). However, it should be emphasized that other studies found no increased cesarean delivery-associated T1DM risk (156, 157).

After delivery, breast milk is usually the first food that enters the gut of the newborn. It has been shown that breastfeeding lowers the risk of developing T1DM compared with formula feeding (158–160). Human milk oligosaccharides protect against autoimmune T1DM development in high-risk individuals (161). As mentioned above, breast milk has a significant effect on the composition of the gut microbiota of infants, and favors the dominance of *Bifidobacterium* because of its specific ability to degrade human milk oligosaccharides (162–164). The decrease in the proportion of *Bifidobacterium*, especially *B. longum* subsp. *infantis*, is temporally associated with the increased incidence of T1DM in childhood (165).

Several studies have reported that antibiotic use increases the risk of T1DM development, in contrast to antiviral or antifungal drugs (166–168). It was shown that exposure to a single antibiotic is not associated with higher diabetes risk, but taking two to five antibiotic courses is associated with an increase in diabetes risk (166). Another study found that the use of broad-spectrum antibiotics during the first 2 years of life is associated with an increased risk of T1DM during the later years of life (167). Moreover, the antibiotic-associated risk for T1DM is influenced by the mode of delivery, being higher in children delivered by a C-section than those delivered vaginally (167). However, it is important to note that other studies did not find a correlation between antibiotic use and T1DM (169, 170).

In agreement with the hygiene hypothesis, newborn infants from less-developed areas who are exposed to a wide range of microbial antigens and therefore receive strong immune signals are protected from diabetes (171).

### Treatment of T1DM

Management of T1DM requires daily administration of exogenous insulin and frequent monitoring of blood glucose levels (172, 173). Despite ongoing technological advances in recombinant insulin, and technologies to deliver insulin and monitor blood glucose levels, the majority of affected patients cannot achieve the recommended glycemic targets (174–177). Particularly in children and young adults, blood glucose control, measured using glycated hemoglobin levels, is typically poor, reaching >8% in the majority when the desired levels are below 7.5% (174). As a result, patients remain at risk of acute and chronic long-term complications associated with the disease.

As multiple islet-specific autoantibodies are found in circulation from a few weeks up to 20 years before the clinical onset of this disease, there is a potential opportunity to prevent or postpone pancreatic β-cell loss. Therefore, other approaches are sought to maintain endogenous insulin secretion or impede the already-developed islet autoimmunity. These approaches include therapy with T_reg_ cells (178–180), treatment with Fc receptor non-binding anti-CD3 monoclonal antibodies (teplizumab and otelixizumab) (181), and antigen-specific peptide immunotherapy that sequesters auto-antibodies (182–184).

### Microbiota in T1DM Therapy

Modulation of the intestinal microbiome of affected individuals seems to be an obvious therapeutic approach for alleviating T1DM. The most promising T1DM treatments, leading to the improvement of the composition and diversity of gut microbiota include strategies such as increasing infant exposure to beneficial bacteria during early life, the transfer of gut microbiota (FMT approach) from presumptively healthy donors to T1DM-prone individuals, and administration of probiotics and prebiotics to replenish or repair the missing bacterial taxa [summarized in a recent review (185)]. It has been shown that probiotics administered in early infancy positively correlated with decreased islet-specific autoantibodies (186). Probiotic treatment improves overall islet function and gut islet immunomodulation and helps to control diabetes (187). Moreover, administration of the natural prebiotic, inulin-type fructans, improve gastrointestinal tract functions with a relative increase in *Bifidobacterium* species compared to the controls (188). In addition, experiments in mouse models of T1DM provide evidence that microbial metabolites, namely the SCFAs (products of fermentation by anaerobic intestinal microbiota), protect genetically-susceptible mice from developing diabetes (189). Nevertheless, the strategies for reshaping gut microbiota require further research exploration, since the “healthy” microbiota composition has not been precisely defined yet. Therefore, only limited conclusions concerning the directions toward modulation of the microbiota can be drawn for now (185).

### Adoptive Therapy for T1DM

As mentioned above, T_reg_ cells are the guardians of immune homeostasis and can inhibit autoreactive immune responses. In healthy individuals, T_reg_ cells migrate to target tissues and suppress inflammation either by direct contact with antigen-presenting cells (such as dendritic cells), metabolic disruption and cytolysis of effector T cells (T_eff_), or secretion of soluble inhibitory cytokines that dampen the function of autoreactive cytotoxic cells (190–192). Importantly, the loss or defect in T_reg_ cell function is implicated in the development of autoimmune diseases like T1DM (179), where the immunosuppressive function of T_reg_ cells is impaired due to the reduction in the cell number, survival, or activity (190). The pathogenesis of diabetes is well mimicked in non-obese diabetic mouse models, where defects in function of T_reg_ cells are also reported (193, 194). Hence, in order to establish immune tolerance, cellular therapies employing thymic T_reg_ cells expanded *in vitro*, under conditions of good manufacturing practice (GMP), are emerging as potentially attractive therapeutic strategies. These cells have been extensively tested for the treatment of T1DM and have been shown to either prevent the development or reverse diabetes in preclinical studies (195, 196), and the first clinical trials with autologous polyclonal T_reg_ cells expanded *in vitro* (178–180, 197, 198). Although no serious adverse effects were reported during the therapy (178, 179), an important concern is the *in vivo* stability of the T_reg_ cell phenotype upon adoptive transfer. This feature of T_reg_ cells is strictly dependent on the stable expression of the master transcription factor FOXP3 (199, 200).

**Figure 3:**
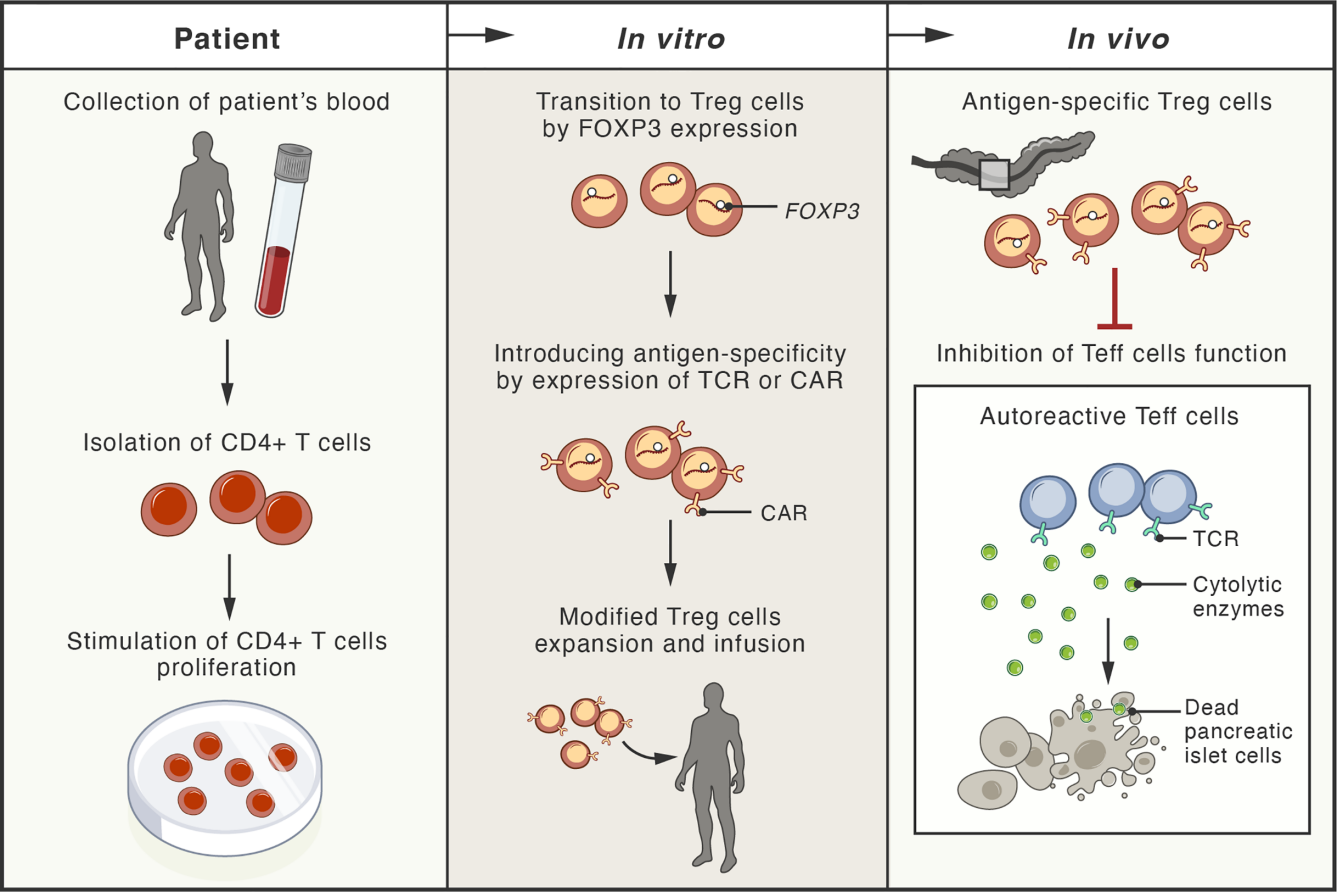
Schema of possible therapeutic approaches with adoptive cell therapies involving genetically modified regulatory T cells. The blood of patient with type 1 diabetes mellitus (T1DM) is used for isolation of CD4^+^ T cells. These cells, expanded *in vitro*, undergo transition to regulatory T cells (T_reg_) by genetic modification, leading to stable expression of the FOXP3 protein (master transcription factor of T_reg_ cells). These cells are further modified by the expression of antigen-specific T-cell receptor (TCR) or chimeric antigen receptor (CAR). Upon infusion, the genetically modified T_reg_ cells localize to the pancreas of the patient, where they inhibit the cytotoxic function of effector T cells (T_eff_), responsible for destruction of pancreatic islet cells.

Recently, a new experimental strategy has been designed to generate genetically engineered T cells from the patient that can function, persist and proliferate like normal T_reg_ cells *in vivo* after infusion, enter the pancreas, and protect the function of islet cells (201). Gene editing techniques based on homology-directed repair were employed in this study. This strategy allows persistent and high expression of FOXP3 from the endogenous locus in patient CD4^+^ T cells, which is sufficient to convert them into functional T_reg_ cells with sustained expression of canonical phenotypic markers and cytokine profiles (201) (**Figure 3**).

The immunosuppressive functions of genetically engineered T_reg_ cells have been demonstrated in a previously characterized murine model of inflammatory disease, where the injection of human T_eff_ cells into immunodeficient NOD/SCID/gamma mice led to extensive T_eff_ cells infiltration into numerous mice tissues followed by an immune response leading to severe xenogeneic graft-versus-host-like disease (202). In such a mouse model, the pre-injection of genetically engineered human T_reg_ cells three days before injection of autologous T_eff_ cells completely suppressed the activation of T_eff_ cells and the development of graft-versus-host disease (201).

The specificity of immunosuppression executed by engineered T_reg_ cells towards autologous pathogenic T_eff_ cells can be enhanced by the expression of either the antigen-specific T-cell receptor (TCR) or the chimeric antigen receptor (CAR) on the surface of the T_reg_ cells (203, 204). Studies using mouse models of autoimmune diabetes have demonstrated that therapy with antigen-specific T_reg_ cells can improve the targeting of the adoptively transferred cells to specific tissues, and allow the specific recognition of common antigens in autoimmune diseases (195, 205, 206). Therefore, the antigen-specificity of T_reg_ cells can improve their suppressive potency as well as the safety and efficacy of T_reg_ cell-based therapies (201, 207).

However, some important considerations concerning the effect of transgenic expression of antigen-specific TCRs or CARs on T_reg_ cell function should be kept in mind. It has been recently shown that the progression of T1DM is associated with reduced diversity of T-cell clones due to the expansion of clones with antigens-specific (mainly proinsulin-specific) TCRs in both autoaggressive T_eff_ and suppressive T_reg_ cell populations. This suggests that some common antigens stimulate the expansion of both T-cell subsets (208). Since the final outcome of the adoptive T_reg_ cell therapy may depend on the balance between T_eff_ and T_reg_ subsets, the therapeutic T_reg_ cells should theoretically be equipped with TCRs or CARs specific to antigens with higher affinity toward T_reg_ cell populations only.

### Controversies and Current ResearchGaps in Adoptive Therapies for T1DM and Potential Future Developments in This Field

The treatment of T1DM remains within the definition of ‘unmet medical need’ as there is no approved therapy stopping the progression of this disease and the only standard of care therapy is substitution with different forms of insulin injections. Undoubtedly, T1DM is the result of an immune-mediated destruction of pancreatic islets, but the primary trigger of this reaction is elusive. Until recently, the autoimmune background has been considered as the most probable mechanism (209), but recent reports also bring up the role of gut-associated microbiome and inflammation (210). The autoimmune background is suggested mainly by the linkage of T1DM with particular HLA haplotypes and organ-specific autoantibodies whose sensitivity and specificity allows them to be used as a laboratory marker of the diagnosis. On the other side, there are a number of autoantigens postulated triggers of autoimmune activation in T1DM, but none of them have been definitively confirmed as a single cause of the disease (211). Moreover, the trials with the use of these autoantigens as agents inducing tolerance in humans failed (212). The inflammatory background also remains unclear. Although the distinct pattern of gut bacteria in T1DM has been described, there is no direct evidence that the disease can be induced by these bacteria or cured by microbiome transplantation (149).

Having no clear target for precisely directed interventions, current attempts of the treatment focus on wider approaches. The most promising studies at the stage of clinical trials in humans cover the use of depletion agents, such as teplizumab (anti-CD3 antibody) (213), or adoptive transfer or induction of tolerogenic cells, such as polyclonal T regulatory cells (197). Briefly, depletion therapies are directed towards elimination (physical or functional) of autoreactive T effector cells, including islet-reactive T cells. The increase in the level of tolerogenic cells aims to switch off unwanted autoantigen presentation and autoimmune response by effector cells, even though the autoantigens are not clearly defined. These approaches come along with the pathogenesis of the disease as it is not only the autoimmunity itself, but rather an imbalance between effector and regulatory subsets, which is regarded as the factor facilitating the onset of T1DM (208). A common feature in this new wave of trials is the attempt to recruit patients who are in a very early stage of the disease, possibly at the asymptomatic phase. Some trials recruit healthy subjects susceptible to the development of the disease, such as members of the families of T1DM patients, offering the therapy as a kind of prophylaxis. These trials give the advantage of a less exacerbated immune process, probable lower numbers of autoantigens involved in the process before substantial epitope spread, and a higher proportion of the pancreas preserved. Novel diagnostic tools already allow us to identify such subjects (214). Scientifically, having insight into such early-phase trials, we might also be able to finally establish the autoantigens that are pivotal for the induction of the disease. It is highly possible that autoantigens change during the course of the disease due to the epitope spread, and particular stages of the disease may require interventions towards different autoantigens. Unfortunately, all these attempts leave behind patients with overt T1DM. It can be assumed that antigen-specific reactions are massive in these cases, and therefore they are much more difficult to harness (215). At the same time, the destruction of the islets is advanced to a high level at this stage. While exogenous insulin remains the only routinely available solution for these patients, they are good candidates for regenerative therapies, but these are slow and tedious to be adopted into clinics (216). Nevertheless, immune interventions might also be necessary here, as the autoimmune reactions are long-term memorized and can reappear many years after the onset of the disease as seen post islet allotransplantation in long-term T1DM patients (217).

## Celiac Disease

CeD is a chronic autoimmune disease caused by a dysregulated immune response to gluten, characterized by remodeling of the small intestinal mucosa and villus atrophy. It is triggered by the ingestion of gluten in genetically predisposed individuals (218, 219). Gluten is a protein component of grains, consumed nowadays in significant quantities. While archeological research has provided evidence of grain consumption as early as 100,000 years ago (220), the more recent (10,000-20,000 years ago) domestication of grains, such as wheat, may have resulted in increased exposure to the gluten protein. Peptides that are the final products of partial gluten digestion can trigger increased gut permeability, gluten trafficking to the lamina propria, innate and adaptive immune response, and tissue damage (221–226). Tissue transglutaminase (tTG) has been identified as the autoantigen in CeD (227).

The genetic susceptibility to CeD is majorly contributed by HLA-DQ2 and/or -DQ8 (219), since the HLA-DQ molecules are responsible for the binding and presentation of gluten-deriving peptides to effector memory T cells that appear to drive the development of CeD (228). Apart from that, at least 39 non-*HLA* genes that predispose certain individuals to the disease have been identified, most of which are involved in inflammatory and immune responses (229). It is estimated that up to 40% of the general population carries the susceptibility genes, but the prevalence of CeD is only about 1% of the general population (230–232) and is higher among women (233, 234). The average age at diagnosis is the scholar age, which is between 6 and 9 years (235), but the disease can occur at any time from early childhood to old age.

Classical CeD is defined as CeD presenting with signs and symptoms of malabsorption: diarrhea, steatorrhea, weight loss, and growth failure. Other intestinal manifestations frequently described are bloating, aphthous stomatitis, alternating bowel habits, constipation, and gastroesophageal reflux disease. Classical malabsorptive symptoms of CeD are more commonly detected in the pediatric population. Up to two-thirds of cases exhibit classical presentations, and atypical symptoms, such as abdominal pain and poor growth, can be the chief complaint in the rest of the cases (236). Extraintestinal manifestations include osteopenia/osteoporosis, anemia, elevation of liver enzymes, and recurrent miscarriages in adults (237). Short stature is the most common extraintestinal manifestation in children, sometimes being the only clinical sign of the disease, and iron deficiency-induced anemia dominates in adults (238–240). Some extraintestinal manifestations are clearly correlated with the severity of intestinal damage (239, 241, 242). Anemia is associated with malabsorption of iron, vitamin B_12_, and folate (243, 244). Growth retardation is caused by nutrient malabsorption (245), and osteopenia may occur due to malabsorption of calcium and vitamin D and the consequent high bone turnover (246). Another specific manifestation of CeD is dermatitis herpetiformis, which is an itchy blistering skin disease typically observed on the elbows, knees, and buttocks, and occasionally in the scalp and upper back (247).

For the diagnosis of CeD, duodenal biopsy plus positive serological tests (anti-tTG antibodies, anti-endomysium antibodies (EmA), and deamidated gliadin peptide (DGP) antibodies) are the gold standard (248, 249). Pediatric patients with high titers (over ten times the cutoff) of anti-tTG antibodies, detectable EmA antibodies, HLA-DQ2/HLA-DQ8 positivity, and symptoms of CeD may skip the duodenal biopsy (230).

### Role of Gut Microbiota in CeD

The incidence of CeD has increased in the past decades, suggesting the role of environmental factors in addition to gluten (250, 251). Patients with CeD present increased intestinal permeability; this may favor gluten sensitization as the characteristic adaptive immune response in patients with CeD takes place in the lamina propria (252). Disruption of the functional epithelial barrier of the intestine by opportunistic pathogens or infections may favor this condition [addressed in depth by a recent review (253)]. In patients with CeD, a shift toward a proinflammatory community and an increase of Proteobacteria and opportunistic pathogens, such as *Neisseria* or *E. coli*, as well as higher bacterial virulence genes have been observed (254–259). *Bacteroides fragilis* strains expressing metalloproteases, which have often been reported in patients with CeD, may lead to increased intestinal permeability, production of immunogenic peptides, and provoke an inflammatory response (260). Increased prevalence of pathogenic bacteria is also observed in the intestines of infants at risk of developing CeD (261).

CeD has been generally associated with alterations in the microbiome composition in both pediatric and adult populations (254, 261–264). Numerous studies have shown that the abundance of certain microbial taxa is significantly different in patients with CeD compared to that in healthy controls. Studies on intestinal biopsies and fecal samples have shown an increased abundance of Bacteroidetes (265) and Proteobacteria phyla (266), and a higher frequency of *Clostridium* (267), *Bacteroides* (265, 268), and *Prevotella* spp (269) in patients with CeD. The abundance of *Lactobacillus* and *Bifidobacterium* spp., on the contrary, is lower than that in controls (265, 270). It may be noted that *Bifidobacterium* can degrade proinflammatory gluten peptides and reduce their immunogenic potential (271, 272). Some lactobacilli have been shown to digest amylase-trypsin inhibitors, which are wheat proteins other than gluten that induce an innate immune response. Moreover, the administration of certain *Lactobacillus* species decreases both the permeability and inflammation stimulated by amylase-trypsin inhibitors (273).

Specific changes in the microbiota of patients with CeD have also been described and associated with poor clinical responsiveness to GFD (274) and the clinical manifestations of the disease (275). For example, compared to patients with other clinical features of CeD, the microbiota of patients with dermatitis herpetiformis is more similar to that of the controls (275).

A recent analysis of the gut microbiota and diet-related metabolites in a large cohort of children affected with CeD revealed specific microbiota signatures for both untreated individuals with new-onset CeD and individuals treated with GFD, with respect to the healthy controls (276). The authors confirmed the overabundance of the microbiota taxa mentioned above, such as *Bacteroides*. Additionally, they detected a lower abundance of *Alistipes* in children with new-onset CeD. The reduction in *Megamonas*, *Ruminococcus*, and *Holdemanella* was recognized as a consequence of the treatment with GFD. Importantly, the authors identified a lower abundance in 11 specific bacterial taxa considered as biomarkers of CeD, with *Clostridium sensu stricto 1* as the most influential one (276).

Changes in the microbiome have also been correlated with alterations in microbiota-derived metabolites (270, 277, 278). It has been shown that patients with CeD have altered fecal SCFAs, the end products of fermentation of dietary fiber by the intestinal microbiota, and increased proteases (254, 279). Duodenal biopsies of patients with CeD show a high proteolytic activity that correlates with the proliferation of protease-producing pathogens such as *Pseudomonas aeruginosa* (254). *P. aeruginosa* cleaves gliadin peptides, whose immunogenicity can be reduced by lactobacilli found in non-CeD controls (254, 273). In addition to intestinal dysbiosis, the salivary microflora, which plays a role in hydrolyzing proline and glutamine-rich peptides, has also been shown to be altered in patients with CeD (280).

It has been shown that environmental factors, such as breastfeeding (281), influences the composition of the intestinal microbiota and may play a role in the development of CeD (282, 283). Oligosaccharides present in human milk enhance the gut barrier integrity by making the epithelium less vulnerable to bacteria-induced innate immunity (284). According to some studies, antibiotic exposure during the first year of life has been associated with an increased risk of developing CeD (285, 286), however, other reports have not found this type of causal association (170, 287). Some studies (234, 288), but not all (289), reported an association between changes in infant feeding practices and an increased incidence or earlier onset of CeD. In particular, a decreased duration of breastfeeding and the discontinuation of breastfeeding upon the introduction of gluten-containing complementary foods to the infant diet has been attributed to the “epidemic” of CeD in a Swedish population of children (234). The protective effect of concomitant breastfeeding and the introduction of gluten might result from the development of oral tolerance to gluten, enhanced by either breast milk-derived immunomodulatory factors or breast milk-stimulated infant gut colonization by beneficial microorganisms (290, 291). On the other hand, large amounts of gluten introduced to the diet of < 2-year-old children increases the risk of CeD (292).

Interestingly, several studies indicate that the *HLA-DQ* genotype may regulate gut colonization (261, 293, 294). The genotype of infants at risk of developing CeD influences the intestinal microbiota composition (294). Compared to control infants, neonates carrying the CeD-predisposing HLA haplotype show increased Firmicutes and Proteobacteria, and reduced Bacteroidetes and Actinobacteria. These patterns persist up to 2 years of age and are correlated with alterations in microbial metabolite production (295). For more details, concerning the association of changes in gut microbiota with risk of CeD development in children and adults, please see recent reviews (296–299).

### Treatment of CeD

The only effective treatment available for CeD to resolve the symptoms associated with the disease and normalize the intestinal villi architecture is a strict gluten-free diet (GFD) (300–302). Generally, the clinical response to this treatment occurs much faster in children than in adults (238, 239). Problems arise when the GFD is not properly followed, which occurs mostly in adolescents (303). Seropositive patients with CeD may gain significant symptom improvement with administration of a mixture of two gluten-targeting recombinant proteases that decrease the immunogenicity of gluten in the small intestine by degrading it in the stomach (304, 305). Another novel oral agent, larazotide acetate, functions by regulating intestinal tight junctions, thereby preventing gluten from reaching the small intestinal submucosa and triggering an immune response (306).

Since the gluten antigen-specific memory T cells are the key players in the pathogenesis of CeD and they are able to persist in patient’s blood and gut for decades (307), new approaches to target such T cells can be considered as promising alternative therapeutic strategies for CeD. Antigen-specific immunotherapy, aimed at tolerance induction, is particularly relevant, since CeD is one of few autoimmune diseases where the antigens and driving pathogenic T cell responses are known (308). In order to induce immune tolerance, gradually escalating doses of disease-relevant antigens can be delivered by systemic or local administration. Different protocols for allergen desensitization have been developed for tolerogenic immunotherapies, with an aim to trigger either reprogramming of antigen-specific T cells into T_reg_ cells or their clonal deletion by inducing cell death. The gluten antigens, delivered with gradual dose escalation, are presented to T cells in a tolerogenic manner. These approaches are either based on the systemic delivery of:

- Vaccines containing gluten-deriving antigenic peptides [such as Nexvax2; clinical trial NCT03644069 (309)],
- Synthetic nanoparticles coated with complexes consisting of antigenic peptides bound to MHC molecules (310),
- Nanoparticles loaded with the antigens, together with immunomodulatory agents [clinical trial NCT03486990 (311, 312)],
- Engineered erythrocytes, loaded with the antigens (313, 314)

or the delivery of antigenic peptides to mucosal tissue, mediated by genetically modified *Lactobacillus lactis* bacteria (315).

**Figure 4:**
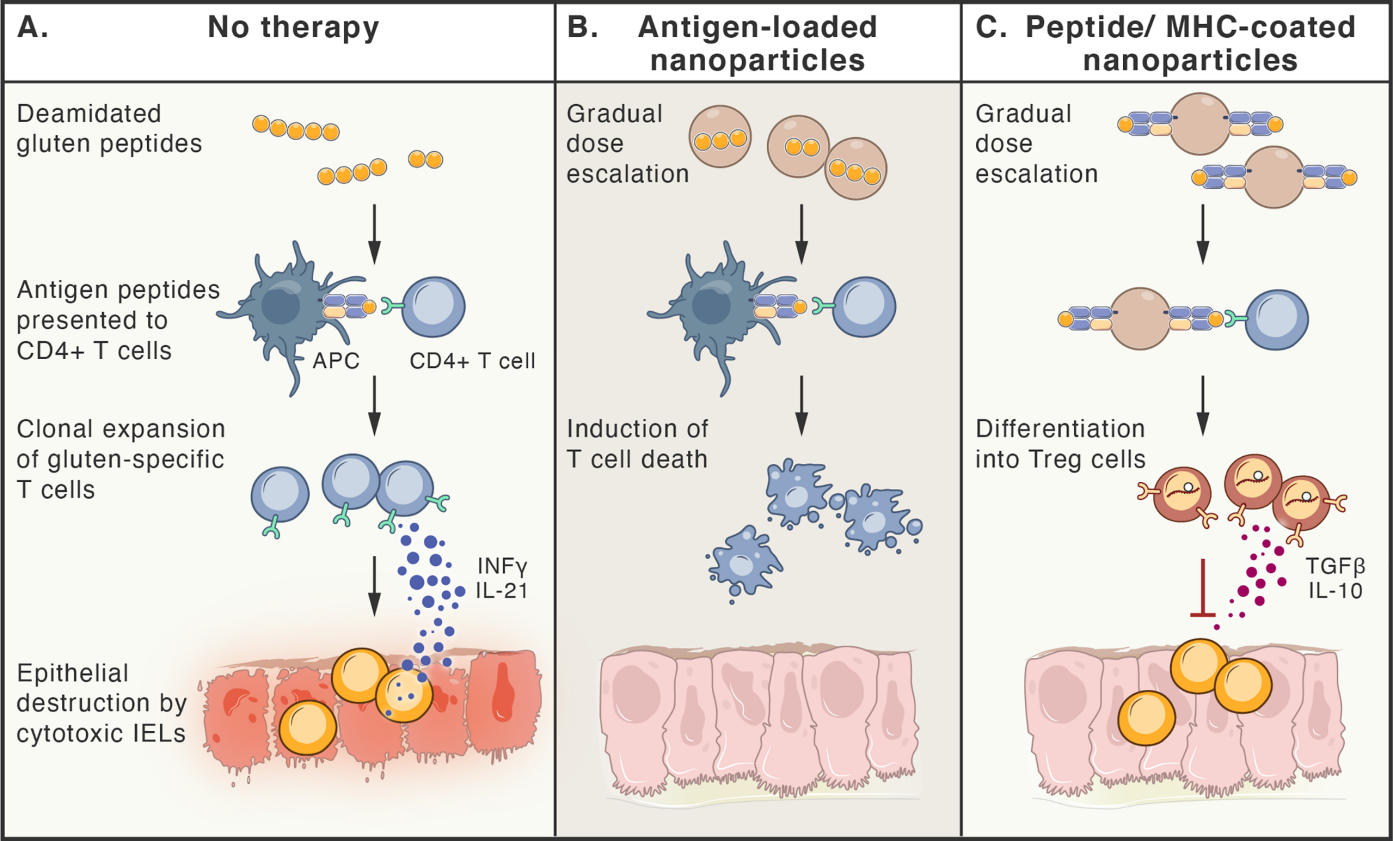
Antigen-specific therapies aiming at tolerance induction in celiac disease. **(A).** Peptides deriving from poorly digested gluten, enter lamina propria, where they are taken up by antigen presenting cells (APCs). Only HLA-DQ2 or -DQ8 molecules are able to bind antigenic gluten-deriving peptides and present them to gluten-specific CD4^+^T cells. These cells become activated, proliferate, release cytokines (e.g. IFN-γ and IL-21) and migrate to the lamina propria, where they induce cytotoxic function of intraepithelial lymphocytes (IELs), that kill epithelial cells. **(B, C).** Therapy-induced tolerance is based on the gradual dose escalation of delivered gluten peptides (e.g. released from nanoparticles or bound to MHC molecules on nanoparticles). Presentation of antigens to gluten-specific CD4^+^T cells in a tolerogenic manner, leads to either death of these T cell clones **(B)** or their differentiation to Treg cells **(C)**, that are able to inhibit immune reaction by multiple ways, including release of TGF-β and IL-10.

Additionally, methods to selectively eliminate disease-driving memory T cells have been tested, including restimulation-induced cell death (such as continuous activation of T cells with antigen and agonists of activating pathways (316), cytokine withdrawal-induced death (317), and selective induction of cell death by interfering with metabolic pathways of activated T cells (318). **Figure 4** summarizes selected approaches for the treatment of CeD.

### Microbiota in CeD Therapy

Compliance with the gluten-free diet (GFD) is difficult (319), and thus, alternative therapies are more feasible (320). The use of probiotics has been suggested as a supplemental treatment for patients with refractory CeD (321). Specific *Lactobacillus* strains have been shown to reduce the immunotoxicity of gluten (322), and probiotic cocktails including *L. rhamnosus* have been shown to improve intestinal barrier function (323). *B. breve* and *B. longum* have been shown to have anti-inflammatory properties in children with CeD (324). In the future, findings on gluten-degrading activities by specific microorganisms may open new possibilities for a probiotics-based complementary therapy of CeD. Many clinical trials involving the use of probiotics to treat and prevent CeD are promising (325), but more studies are needed to better understand the exact mechanisms linking dysbiosis and probiotics with the onset and development of this disease. The studies comparing the fecal samples and duodenal biopsies, obtained from patients with CeD versus healthy individuals, have shown a severe alteration of gut microbiota (270, 326, 327). When patients with CeD were treated with GFD, the increased concentration of bacteria was reduced to that in the healthy population, which suggests the influence of diet on intestinal microbiota. However, in most studies only partial modulation of the microbiota was observed on the GFD (328, 329). *In vitro* studies to assess the use of probiotics in the treatment of CeD, have demonstrated that selected *Lactobacilli* strains, when added to sourdough fermentation, lyse the proline/glutamine-rich gluten peptides, reduce the gluten concentration to <10 ppm (gluten-free), and decrease their immunotoxicity (330). Four strains of *Lactobacilli* (*L. ruminis, L. Johndoni, L. amylovorus, L. salivaris*), capable of degrading and reducing the immunotoxicity of gliadin peptides, were identified from the proximal gastrointestinal tract of pigs (331). In a study including 20 patients with CeD, receiving hydrolyzed wheat gluten bread (containing *Lactobacillus alimentaris, L. brevis, L. sanfranciscenis, L. Hilgardi*) for six days, no significant increase in IFN-γ responses, compared to healthy controls, was found (332). In another study, that challenged CeD patients in remission for 2 months with *Lactobacilli* predigested gluten, no worsening of symptoms, histological structure of the small intestine or serological marker was found (333). This outcome suggests that *Lactobacilli*-derived peptidase was capable of completely degrading gluten and reducing its immunotoxicity in CeD (334). These studies indicate that the addition of probiotics, rich in *Lactobacilli* spp., may alleviate the consequences of accidental or contaminant gluten exposure (333). Dysbiosis in CeD is associated with abnormal tight junctions and increased intestinal permeability. Lindfors *et al.* studied the effects of probiotics on human colon cells and demonstrated that *B. lactis* decreased intestinal permeability, and the effect was dependent on the probiotic’s dose (272). Moreover, *Bifidobacteria* downregulated *in vitro* proinflammatory cytokines production induced by gliadins (271) or by fecal samples from patients with CeD. This suggests that *Bifodobacterial* strains can reverse the effects of CeD-associated microbiota (335). *Lactobacillus rhamnosus GG* strain decreased gliadin peptide-induced changes in intercellular junction proteins and gliadin-induced enteropathy in rats. Similar beneficial effects of probiotic *B.longum* CECT 7347 were observed in the small bowel of weaning animals fed gliadin. These observations may suggest that early administration of probiotics can have a protective effect on the intestinal mucosa (336). All the above-mentioned studies indicate a beneficial effect of probiotics on digestion of gliadin peptides, intestinal barrier, and immune system, as well as on intestinal mucosa (337–339).

## Inflammatory Bowel Disease

IBD is a group of chronic, relapsing, and remitting inflammatory conditions that primarily affect the gastrointestinal tract (340–342). Traditionally, it has been divided into Crohn’s disease (CD), ulcerative colitis (UC), and IBD unclassified (IBDU). It begins most commonly during adolescence and young adulthood, as up to 25% of IBD patients are below 18 years of age (343–346).

The cause of IBD remains poorly understood; however, research shows the involvement of genetics, microbiome, environment, and immune system (347). The disease has a complex multifactorial etiology with genetic defects in pathways associated with the immune system, epithelial barrier, and infections (57, 347, 348). Interestingly, among numerous genetic loci associated with IBD risk, there is also the *PTPN22* gene (349), one of the previously mentioned risk factors for T1DM. In contrast to diabetes, the autoimmunity-associated polymorphic variant of the PTPN22 protein seems to be protective against IBD risk, an effect associated with alterations in the gut microbiota composition in humans (350) and mouse models of colitis (351). There is an overlap in the genetic susceptibility to CD and UC, with patients frequently having subtypes of both diseases in their family history (347, 352, 353).

CD and UC have typical features that can aid diagnosis. CD can occur from the mouth to the anus, and it is patchy, transmural, granulomatous, and may have stricturing or penetrating (fistulating) features (354, 355). In addition, 20% of children with CD have perianal involvement, such as skin tags, fissures, fistulas, and abscesses (356). Conversely, UC is a disease of the colonic and rectal mucosa that usually does not lead to fibrotic strictures or perianal disease (357). IBDU, which does not fit into the other groups, is more common in children (358). Extraintestinal manifestations of IBD include dermatologic conditions, such as erythema nodosum and pyoderma gangrenosum, arthritis, growth failure, osteoporosis, and anemia. Approximately 20% of children present with extraintestinal manifestations of IBD, such as growth failure, anemia, and perianal disease, as the only initial features (359).

For IBD diagnosis, intestinal endoscopy with biopsy remains the standard (355). Examination of the stool is done for inconspicuous blood and pathogens. Fecal calprotectin, a neutrophil-derived protein with elevated concentrations during intestinal inflammation, is a useful biomarker (360, 361).

Breastfeeding is known to protect against the development of IBD, with greater benefits accruing from a longer duration of breastfeeding. In addition, high fiber and fruit intake is associated with a decreased risk for CD, and a high intake of vegetables is associated with a decreased risk for UC. Diets high in fats and meat are associated with an increased risk of IBD (95, 362, 363).

### Role of Gut Microbiota in IBD

Multiple lines of evidence support the important role of the microbiota in the initiation and progression of IBD (364), highlighting reduced microbial diversity in the guts of both pediatric and adult patients (365–367).

Previous methods for pathobiont discovery in IBD patients focused on identifying intestinal bacteria bound to IgA (368, 369). Currently, nucleic acids sequencing analysis of IgG-bound microbes is considered a more clinically relevant method (370), since in IBD patients, the level of IgGs in the gut lumen is elevated (371), and IgGs seem to be more specific toward pathogens than IgAs (372). Analysis of the microbiome in pediatric IBD patients revealed increased IgG binding by invasive strains, such as *Burkholderia cepacia*, *Flavonifractor plautii* and *Rumminococcus* sp., while IgG binding of non-invasive *Pseudomonas protogens* was reduced (370). Other studies reported alterations in certain genera of the phylum Firmicutes and increased abundance of *Enterobacteriaceae* species in adults affected by IBD (373, 374). Some studies have also shown changes in *Bacteroides* spp (375). These changes are generally more pronounced in patients with CD than in patients with UC (376). Numerous other disease-specific changes in the microbiome have been recently reported in IBD patients (377, 378). Several pathogens, including *S. enterica*, *Shigella flexneri*, *Yersinia enterocolitica*, and *V. cholerae* may participate in IBD pathogenesis because they produce mucin-degrading enzymes (379–382), and they may break down the intestinal mucosal barrier that reduces the contact between microorganisms and the epithelial cell surface. In this manner, pathogens may increase the susceptibility of IBD development (383, 384). In addition, decreased numbers of SCFA-producing microorganisms, such as *Clostridium* spp. and *Faecalibacterium prausnitzii*, have been observed in patients with IBD (373, 385–387). The decreased abundance of *Faecalibacterium prausnitzii* in the ileum is associated with an increased risk of postoperative recurrence of ileal CD and endoscopic recurrence at six months (385). *Roseburia* spp. and *F. prausnitzii*, members of the Firmicutes phylum, are among the most beneficial microbes (385, 388). Besides producing SCFAs by fiber fermentation, these microbes also secrete several anti-inflammatory metabolites (389). SCFAs, particularly butyrate, promote the development of T_reg_ cells and mucus production to downregulate inflammatory signaling pathways (390, 391).

Another feature of IBD is the reduction of the tryptophan metabolite, indoleacrylic acid, produced by several *Peptostreptococcus* spp., which also promotes the function of the mucosal barrier and reduces inflammatory responses (392, 393). Some great reviews addressing the complex interplay between important dietary factors and microbiota profiles in health and IBD have been recently published (394–396).

Although the differences in microbiota composition may indeed reflect IBD-specific changes, one must keep in mind that the microbiota composition exhibits significant heterogeneity between presumptively healthy individuals, even within the same person, when it is assessed at different time points (366, 397). This conclusion underlines the need for large cohort studies, that may differentiate between disease-dependent and –independent, as well as disease activity-dependent changes in microbiota composition. A recent large cohort longitudinal study ranking the main factors affecting microbiota variance in IBD patients listed geographic location, CD diagnosis, history of surgical resection, consumption of alcohol, presence of UC, medications taken, and dietary habits as the most significant (366). A recent machine-learning analysis uncovered an additional level of complexity in the relationship between the gut microbiome and disease activity, reporting microbiome influence on gene regulation (transcriptomic profile) of the host in large groups of patients with colorectal cancer, IBD and related Irritable Bowel Syndrome. Moreover, the regulation of different host signaling pathways by the gut microbiota seems to be disease-specific [https://doi.org/10.1101/2021.03.29.437589 and (398)].

### Treatment of IBD

Current therapeutic strategies focus on treating IBD relapses and prolonging remission (340–342). For the induction of remission, exclusive enteral nutrition, corticosteroids, or anti-TNF-α antibodies (infliximab and adalimumab) are used (399, 400). Exclusive enteral nutrition, which is a nutritionally complete elemental and polymeric formula diet that contains no solid food, is the first-line option for induction of remission in pediatric CD (341), because it is as effective as corticosteroids in inducing remission, but without the side effects associated with corticosteroid therapy (401–403). Once remission has been established, it is maintained with the following agents: 5-aminosalicylate, thiopurines or anti-TNF-α antibodies. New-generation monoclonal therapies include vedolizumab and ustekinumab. Surgical intervention is often required in both UC and CD, as seen in 10% and 25% of children prior to the age of 18 years, respectively (404, 405). Unfortunately, there is no permanent cure for the disease. Medications currently available are extremely effective, but have significant potential toxicity, including increased infection risk, steroid toxicity, and increased risk of malignancy.

**Figure 5:**
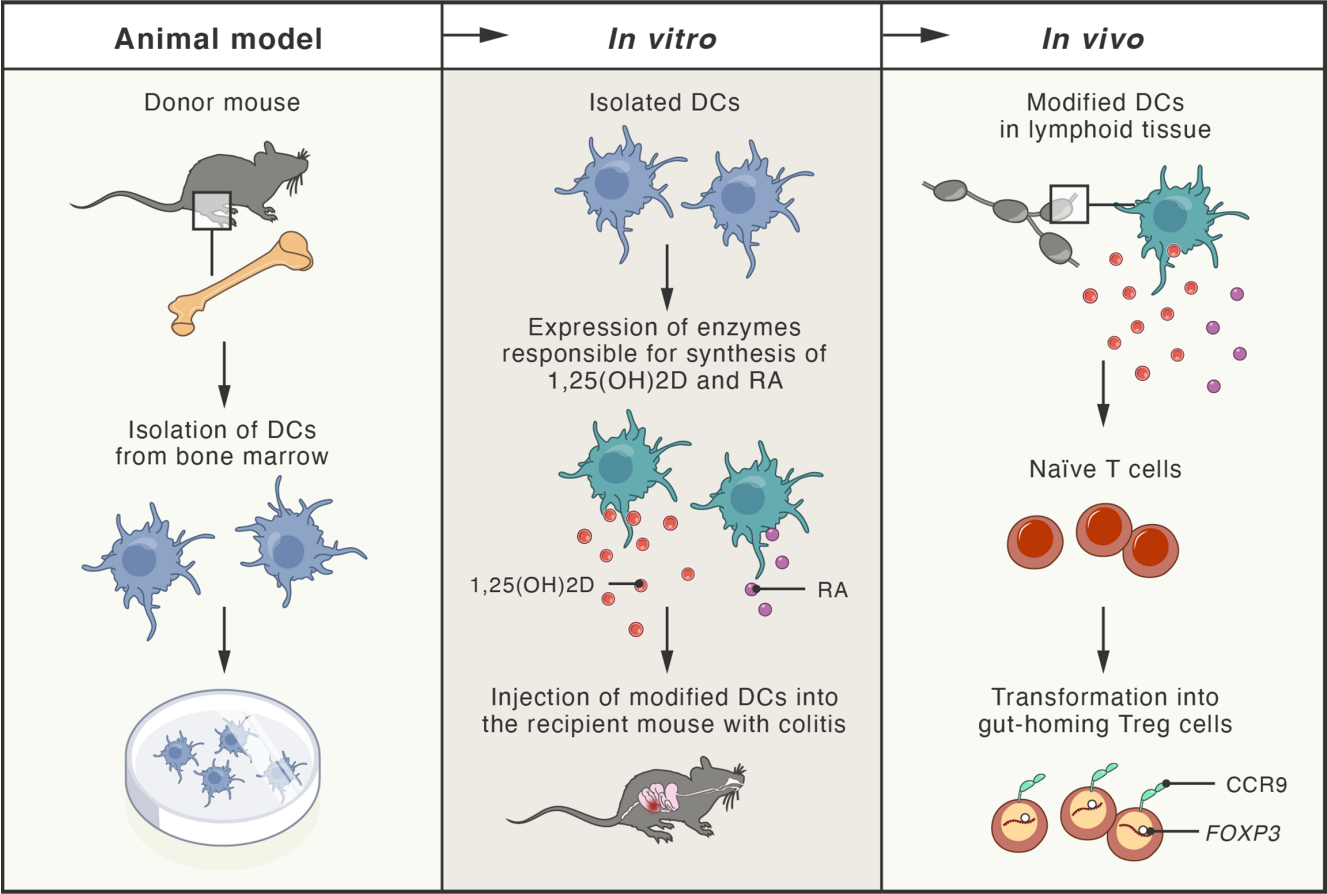
Schema of new therapeutic approach tested in mouse models of colitis. Dendritic cells (DCs) obtained from the bone marrow of donor mice were genetically modified to express enzymes responsible for production of 1,25-dihydroxyvitamin D (1,25(OH)_2_D; active vitamin D metabolite) and retinoic acid (RA; active vitamin A metabolite). Modified DCs were transferred to the recipient mice with experimentally-induced colitis. In peripheral lymphoid tissues, 1,25(OH)_2_D and RA (produced by the modified DCs), induced naïve T cells transformation into gut-homing regulatory T cells (Treg), by induction of FOXP3 and CCR9 (gut-homing receptor). T_reg_ cells efficiently homed to the intestine, where they inhibited inflammation and colitis.

Since the loss of immune homeostasis due to defects in the number and suppressive function of T_reg_ cells has been documented in IBD (406), alternative therapies employing adoptive T_reg_ cell transfer have been explored in numerous studies using mouse models of colitis (407–410). In particular, T_reg_ cells secreting anti-inflammatory cytokine IL-10 have been shown to partially prevent the development of the disease (411, 412), while the disruption of IL-10 expression in T_reg_ cells, residing in the intestinal mucosa, led to the development of spontaneous colitis in mice (413). Therefore, numerous approaches for the expansion of human T_reg_ cells *in vitro*, under GMP conditions, have been explored, including application of rapamycin and an agonist of retinoic acid receptor (414–416). These agents are also being used in ongoing phase I/IIa clinical trials (TRIBUTE; NCT03185000), initiated for the evaluation of therapeutic adoptive transfer of T_reg_ cells in patients with CD. Another successful strategy included the injection of expanded T_reg_ cells (treated with retinoic acid receptor agonist) together with recombinant IL-2. In this study, an immunodeficient mouse model of CD with subcutaneously implanted human intestine tissue was used (417). Patients-derived T_reg_ cells were expanded *in vitro* and adoptively transferred to the mice, where they efficiently homed to the human small bowel. *In vitro*-expanded T_reg_ cells were also effective in suppressing the function of T_eff_ cells isolated from inflamed Crohn’s mucosa (417). The proper tissue-specific migration and gut homing of T_reg_ cells are critical factors influencing the success of T_reg_ cell-based therapies. Integrin α4/β7 and CCR9 protein have been identified as gut-homing receptors that can be simply induced during T_reg_ cells expansion by addition of retinoic acid (RA) to the cell culture (418). In another interesting approach, researchers engineered dendritic cells (DCs) to produce high concentrations of both RA and 1,25-dihydroxyvitamin D (1,25(OH)_2_D). Since 1,25(OH)_2_D induces expression of FOXP3 and IL-10, while RA stimulates expression of gut-homing receptors in T cells, the approach led to the induction of T_reg_ cells *in vivo*, in peripheral lymphoid tissues, and subsequent homing of T_reg_ cells in the intestine followed by stable suppression of intestinal inflammation in a mouse model of colitis (419) (**Figure 5**).

An important consideration in therapeutic strategies employing T_reg_ cells is the antigen specificity of their TCRs. The polyclonal population of T_reg_ cells, residing in the intestine, relies on the broad reactivity of its TCRs, which recognize a broad spectrum of microbiota-derived antigens, increasing the likelihood of TCR activation and proper maintenance of immune tolerance and intestinal homeostasis. Not surprisingly, the limited TCR repertoire in mice results in the development of spontaneous colitis (420). On the other hand, for the therapy of experimental colitis in mice, the antigen-specific T_reg_ cells are more potent than polyclonal T_reg_ cells (421, 422). An interesting approach with such antigen-specific T_reg_ cells has been reported in phase I/IIa clinical trials for adults (CATS1 study) using T_reg_ cells that specifically recognize a common food antigen of ovalbumin (423). The ovalbumin-enriched diet, in the form of a meringue cake, was used by the patients with refractory CD to facilitate the local activation of therapeutic T_reg_ cells. This approach led to partial remission with mild adverse effects.

### Microbiota in IBD Therapy

Administration of probiotics, including *Lactobacillus* or *Bifidobacterium* spp., has been successful in some bowel disorders (424). Oral treatment with the probiotic *E. coli* Nissle 1917 has been shown to maintain disease remission in patients with UC (425). Probiotic *E. coli* Nissle 1917 can secrete microcins with antimicrobial activity, and suppress competing *Enterobacteriaceae* that may exacerbate gut inflammation (426). A mixture containing eight probiotic organisms has been proven to be beneficial as an addition to standard therapy for patients with UC (427). However, at present, probiotics are not recommended in the guidelines on the management of UC because of insufficient high quality evidence (428). It has also been shown that fecal stream diversion decreases inflammation in ileal CD (429). Despite some encouraging data, FMT (described in more details in the next paragraph) remains an investigational treatment that is used only in clinical trials (430). Moreover, antibiotics can induce remission and prevent relapse in patients with IBD (431, 432). However, despite the benefits of antibiotics for the treatment of CD and UC, their use in modifying the microbiota is limited by their inability to selectively eliminate pathogenic bacteria without affecting beneficial microorganisms, particularly with prolonged or repeated courses (433, 434). Currently, antibiotics are recommended to treat diseases complicated by infection (abscesses, bacterial overgrowth, *Clostridium difficile*) or perianal fistulising disease (428). Exclusive enteral nutrition, which is the dietary intervention used in CD, quickly alters the microbiota composition and effectively reduces intestinal inflammation in pediatric patients (435). The mechanism by which this diet induces remission in CD remains unclear, but it may promote the growth of beneficial microorganisms or the depletion of pathobionts. Unfortunately, there is still a lack of knowledge about the identity of IBD-causing pathogens that trigger inflammation in genetically susceptible individuals.

The outcome of new therapeutic strategies, such as adoptive therapy with T_reg_ cells, is likely to be modulated by the intestinal microbiota, which represents a substantial antigen load in the gastrointestinal tract. In healthy individuals, T_reg_ cells residing in the intestinal lamina propria provide tolerance towards the gut microbiota and their suppressive capacity is enhanced during intestinal inflammation (436, 437).

## Controversies and Current Research Gaps in Modulation of Microbiota Composition and Potential Future Developments in This Field

Recent advances in microbial genomic sequencing and other biology techniques allow for novel insights into the potential contribution of the gut microbiota to health and diseases (438). Consequently, changes in the composition and functionality of the gut microbiota have been found in an increasing number of diseases (439–442). However, it remains unclear whether dysbiosis is a cause, a consequence, or incidental to the disease. FMT is currently gaining increasing clinical and research importance. At present, FMT is recommended to treat recurrent *Clostridioides difficile* infection (CDI), but there is a growing number of ongoing trials exploring its other potential therapeutic indications (443). One of them is IBD. An altered microbiome has been described as one of the factors contributing to the pathogenesis of IBD, but it is still unclear whether this is a cause or effect of the gut inflammation in Crohn’s disease (CD) and ulcerative colitis (UC) (433, 444). The safety and efficacy of FMT in patients with both IBD and CDI have been assessed prospectively in the NCT03106844 study. Currently, the research continues to advance toward exploring this treatment for IBD. Four randomized controlled trials, which assessed the use of FMT in UC, have been published, and in three of them a significantly increased rate of both clinical and endoscopic remission in UC patients, receiving FMT compared to those receiving placebo, were reported (445–448). In a recent Cochrane systematic review, an overall remission rate at week 8, across these four studies was 37% (n=52/140) in patients receiving FMT, compared to 18% (n=24/137) in those receiving placebo (relative risk 2.03; 95% confidence interval: 1.07–3.86) (449). The highest rates of steroid-free response and remission were reported in the single study that used anaerobic conditions for FMT preparation, suggesting that this can be a relevant factor. One study demonstrated better outcomes with one donor of the stool than with other donors. This observation may indicate that donor selection may be much more important in UC than in CDI. However, microbial characteristics for an optimal donor in IBD have not been well defined, and the hypotheses mentioned above require further studies. Since the role of the gut microbiota in the pathogenesis of UC is under intense investigation, a future study will focus on exploring whether successful donors have a gut microbiota particularly enriched in specific microbial strains that are absent in the gut in UC. Overall, the existing evidence suggests that FMT may be potentially useful for treating mild to moderate UC. When it comes to CD, only studies on relatively small adult and pediatric cohorts have been published. A recent meta-analysis of eleven studies, including four case reports and seven cohort studies, reported an overall 50.5% (n=42/83) rate of clinical remission in CD patients receiving FMT (450). However, the heterogeneity of disease activity and FMT administration protocols of the included studies limited the drawing of conclusions. A double-blind randomized controlled trial, evaluating the efficacy of FMT in adults with CD is ongoing (NCT03078803).

The FMT can also be considered a potential therapeutic approach in T1DM. The composition of the gut microbiota in T1DM-affected children or individuals at high risk of developing T1DM is different from that in healthy individuals (138, 146, 451–453). Additionally, in many cases, changes in microbiota composition have been detected before the first symptoms of T1DM (146, 452), suggesting a functional association between changes in microbiota and the disease onset. In a recent trial (NTR3697), the positive health effects of FMT in ten young patients (18-30 years old) with new-onset T1DM were detected (454), providing a basis for future clinical trials. On the other hand, standardization of transferred microorganisms used in the FMT approach may be necessary, and further profiling of microbiota composition in “effective” donors is urgently needed. The data published so far indicate that adverse events of FMT occur in about 20% of patients, however, most of them are mild (455). Only one case of serious adverse events, myasthenia gravis, has been reported (456). According to current ECCO/ESPGHAN guidelines for the medical management of pediatric CD, both probiotics and FMT are neither recommended for induction nor remission maintenance (457). It seems that in contrast to CDI, frequent transplantation is necessary for FMT to be effective for any chronic disorder. It has been reported that improved microbial diversity can persist for several weeks, but does not persist after 1 year (448, 458). The pathophysiology of chronic illness is very complex and will likely require more long-term FMT or controlling inflammation in the recipient intestine to facilitate the engraftment [broadly reviewed in Ref (459)].

## Discussion

Chronic inflammatory disorders are often managed with symptomatic therapy, and eventually lead to multiple downstream sequelae (460–462). Despite many similarities between this group of diseases in children and adults, pediatric onset often presents with atypical features (460, 463, 464). Pediatric patients are particularly vulnerable to adverse effects of drugs because the number of clinical trials in this group of patients is limited, and drug absorption and metabolism are more variable and less predictable (465, 466). An additional challenge in pediatric care is achieving normal growth, puberty, and access to education. The impact of drugs on child growth and development is still not fully understood. Further studies are needed to better understand the etiology and pathogenesis of these inflammatory disorders, and to identify preventive strategies, alternative or complementary therapies, and prevent complications. The interaction between genetic and environmental factors is crucial in the development of chronic inflammatory disorders (467). Continued research on the specific role played by the gut microbiota and the complex interactions between microorganisms and the host will help to address these issues better (468). Data on the importance of the interactions between microbiota and the human immune system, the crosstalk between various species of microbes, and details on the communications between various organs and their microbiota is still lacking (469, 470).

Some novel technologies employing genetic engineering of immune cells, as well as adoptive transfer of modified patient immune cells to treat the disease, are being extensively tested in laboratories and clinical trials (471–473). The therapies involving engineered T_reg_ cells that are expected to specifically suppress the proliferation and production of inflammatory cytokines by cytotoxic T_eff_ cells in the pancreas and stop the destruction of pancreatic islet cells, or at least slow the progression of T1DM (201), are particularly promising. However, several considerations should be kept in mind when using these therapies, including the antigen specificity of the transferred engineered immune cells, and their interactions with the gut microbiota that may affect the therapy outcome in multiple ways (474, 475). Advances in scientific research must always be seen in the context of clinical care, outcomes, and prognosis for children with diseases, such as T1DM, CeD, and IBD.

The gut microbiota should be certainly considered as one of the potential factors that can modulate the responses to immunotherapies. The outcome of new therapeutic strategies, such as adoptive therapy with T_reg_ cells, is likely to be modulated by the intestinal microbiota, which represents a substantial antigen load in the gastrointestinal tract. In healthy individuals, T_reg_ cells residing in the intestinal lamina propria provide tolerance towards the gut microbiota and their suppressive capacity is enhanced during intestinal inflammation (436, 437). However, since adoptive cell transfer is a relatively novel therapy for autoimmune diseases, currently, little is known about the influence of gut microbiota on the outcome of adoptive therapy with T_reg_ cells. On the other hand, progress has recently been made in understanding the role of gut microbiota in modulating CAR-T cell outcome in cancer therapy and demonstrated that gut microbiota can influence the balance between activity of effector CAR-T cells and suppressive activity of T_reg_ cells [reviewed in Ref (476)]. By analogy, since the predominant function of adoptively-transferred T_reg_ cells in therapy of autoimmune diseases is to control activity of autoreactive effector T cells, one can expect that the gut microbiota may also influence this process.

## Concluding Remarks

Undoubtedly, the microbiota significantly influences health status. Although the origin of diseases described in this review differ, an improper balance in microbiota content seems to be common to the progression of all of them. The inflammation behind untolerated or an altered composition of the microbiome is currently attributed to the syndromes closely associated with the alimentary tract but also with many other diseases. The discovery of the interaction between saprophytic bacteria and our tissues has increased our understanding of the pathogenesis of many diseases. It was once overwhelming to think that the gut bacteria might contribute so heavily to our daily well-being. Fortunately, this knowledge allows us to modulate the microbiome, which has become a novel way to treat many diseases. The awareness that the microbiome influences us continuously is also acknowledged with many novel therapies, and notably in those that are disease-modifying. The microbiome as a factor affecting the efficacy of biologics or cell therapies is now considered a good and cheap way to improve such responses. Bacteria have been our commensals for a long time, and the reciprocal benefits of this symbiotic relationship should not be a surprise. Further exploration of the role of the microbiome in our life will definitely give us many novel tools for disease treatment.

## Author Contributions

All authors participated in manuscript preparation, including text (AT, BP, JK, PT, AH) and figures (AH, AT, BP) preparation. All authors contributed to the article and approved the submitted version.

## Funding

This work was supported by the National Science Centre (NCN, Poland) with grant no: 2016/23/B/NZ5/02622.

## Conflict of Interest

PT is co-inventor of patents related to presented content and stakeholder of POLTREG venture (company commercializing therapy based on T_reg_ cells). Medical University of Gdansk received payments and royalties for the license to presented content.

The remaining authors declare that the research was conducted in the absence of any commercial or financial relationships that could be construed as a potential conflict of interest.

## Abbreviations

APC, antigen-presenting cell; CAR, chimeric antigen receptor; CD, Crohn’s disease; CDI, *Clostridioides difficile* infection; CeD, celiac disease; DCs, dendritic cells; DGP, deamidated gliadin peptide; EmA, anti-endomysium antibodies; FMT, fecal microbiota transplantation; GF, germ-free; GFD, gluten-free diet; GMP, good manufacturing practice; GALT, gut-associated lymphoid tissue; HLA, human leukocyte antigen; IBD, Inflammatory bowel disease; IBDU, IBD unclassified; Ig, immunoglobulin; IFN-γ, interferon-γ; IL, interleukin; NK, natural killer; RA, retinoic acid; SCFA, short-chain fatty acid; T1DM, type 1 diabetes mellitus; TCR, T-cell receptor; T_eff_, effector T cells; TNF-α, tumor necrosis factor-α; T_reg_, regulatory T cells; tTG, tissue transglutaminase; UC, ulcerative colitis.
